# Predictors of quality of life of people receiving intensive community-based care

**DOI:** 10.1007/s11136-015-1093-5

**Published:** 2015-08-30

**Authors:** P. M. J. Emmerink, D. P. K. Roeg

**Affiliations:** Tranzo Department, Tilburg University, Warandelaan 2, 5000 LE Tilburg, The Netherlands

**Keywords:** Assertive community treatment, Cross-sectional studies, Epidemiological determinants, Quality of life, Social determinants of health

## Abstract

**Purpose:**

Intensive community-based care (ICBC) is a home-treatment approach aiming to support people ‘living in the community’ with severe psychiatric and addiction problems. Although subjective quality of life (SQOL) is an increasingly important outcome measure in health care, little is known on ICBC clients’ SQOL.

**Methods:**

Clients of three ICBC teams (*N* = 523) participated in the study. Upon intake, clients filled out a SQOL measure and indicated whether they had a good friend, partner, and children, as well as their experiences with crime. Professional caregivers filled in a measure on problem severity.

**Results:**

Regression was used to examine to what extent the included variables contributed to explaining variance in ICBC clients’ SQOL. Determinants in the model significantly predicted client SQOL and explained 37 % of the variance. ‘Symptomatology’ (depressive symptoms) and ‘social problems’ (living conditions) negatively influenced the SQOL, while having a partner, a good friend, and an overall lower problem severity positively influenced SQOL.

**Conclusions:**

SQOL among ICBC clients is related to psychopathology, in contrast to previous knowledge. It is dependent upon symptom specificity, living conditions, and social circumstances and therefore presumably on program characteristics. This study provides insight into well-being among ICBC clients and is therefore relevant to involved healthcare professionals.

## Introduction

Intensive community-based care (ICBC) is a form of non-committed care that is offered to marginalized individuals in the community. The programs, that were originally developed in the early 1970s as a response to the closing down of psychiatric hospitals, provide a home-treatment-based approach that aims to support people with severe psychiatric or addiction problems with ‘living in the community.’ In this way, the programs aid in maintaining contact between these people and health services. ICBC programs with international recognition are, for instance: assertive community treatment (ACT) or assertive outreach [5]. Whereas originally ICBC was mainly focused on the reduction in psychiatric hospital admissions, improving client income, and housing situations, nowadays, the goal of improving clients’ subjective quality of life (SQOL) has become more important [1–5].

However, although pivotal to most contemporary ICBC programs, a minority of the studies on ICBC has focused on subjective outcomes, such as SQOL [6–10]. The use of SQOL as an outcome measure in studies in the field of mental health care has greatly increased since the 1990s, and the consensus has been achieved that SQOL has a number of advantages over clinical outcome measures. SQOL measures are more comprehensive than focusing on symptomatic cure only; they take into account the own perception of the individual on his or her life; they are holistic in capturing more life aspects compared to objective or clinical outcomes; and they provide an opportunity to uniformly compare across populations and interventions [11, 12]. Furthermore, SQOL concurs with current mental healthcare objectives in that it takes into account several other life domains important to mental health, e.g., the social environment and mental well-being. These domains have previously been shown to be highly relevant to individual recovery [12, 13].

Despite its importance, only limited knowledge is currently available on the determinants of SQOL for people with (severe) mental health problems. One previous study, in which the SQOL of clients in general mental health care with different diagnoses was compared, showed that persons with schizophrenia had more favorable SQOL scores than those with mood and neurotic disorders. Furthermore, in all diagnostic groups, older patients, those in employment and those with lower symptom severity scores, showed higher scores on SQOL [14]. Specifically for persons attending ICBC services, one other study found that SQOL was only minimally associated with psychopathology [11]. In the same study, demographics, diagnosis, disability, function, and service use were only weakly related to SQOL. The difference in results between these two studies might be explained by differences in the type of problems that clients experience in other life areas that have not been thoroughly examined. Therefore, a more comprehensive examination of the determinants of SQOL, including the type of problems experienced in other life areas, is warranted. On a side note, the importance of taking into account the level of SQOL upon entrance into a mental healthcare program is apparent from a recent study on clients attending so-called interferential care (a type of ICBC) in the Netherlands, which showed that their SQOL was relatively low at entrance into the program, compared to clients entering ACT ICBC programs in the Netherlands and other ICBC programs in the UK [15]. Secondly, the problem severity of the clients needs to be taken into account as well: In that same study, the problem severity in the ‘interferential care’ group was very high (compared to clients using mental health day care services). Moreover, the life areas that caused the most severe problems differed between the ‘interferential care’ ICBC and the regular mental healthcare group. In comparison with the regular mental healthcare group, the ‘interferential care’ ICBC service users scored particularly poor on social problem areas, including housing, self-care, employment/leisure activities, social relationships, and substance use [15].

The present study aims to add to the understanding of SQOL determinants of clients entering ICBC programs by taking into account problems in different life areas as well as controlling for problem severity upon entrance into the mental healthcare program. These determinants will be investigated by comprehensively including demographics, social environment, and specific problems into a single research design, with SQOL as the outcome measure. Using data that are routinely collected at intake by the ICBC teams, the findings will be able to provide the involved mental healthcare professionals with clear guidance in improving their clients’ SQOL.

## Methods

### Setting the scene

The Netherlands employ many social services in order to produce a social safety net for those vulnerable people in the society, i.e., those who are less able to take care of themselves. Social security is thus in place for groups like the elderly and those unable to work. Additionally, there are some services that are especially designed to help individuals marginalized from society, such as people who are mentally, socially, behaviorally, or physically incapacitated due to severe psychological or social problems. This includes people with psychiatric or addictive problems. Therefore, it is particularly difficult for an individual in a country such as the Netherlands to end up being unknown to care services and ICBC ‘interferential care’ teams can be perceived as the very last safety net. ‘Interferential care’ actively reaches out to individuals previously unknown to care services, who have now in some way been referred to an interferential care team [16, 17]. The population includes clients with a combination of severe problems including mental problems, addiction, social, and financial problems and excludes those with a diagnosed indication for a mental healthcare disorder (ACT teams are set up for this latter group).

### Sample

The study group consisted of clients, over 18 years of age, entering interferential care programs in three different regions in the south of the Netherlands during the inclusion period of November 2008 to April 2011. The included programs were provided by interdisciplinary teams and shared a number of characteristics with ACT as well as with brokerage model programs [18] in that the teams provide full services during a number of months but aim at referral to regular healthcare services afterward. Services provided during the interferential care consist mainly of practical support. Involved care professionals come from different organizations (i.e., mental health care, addiction care, social work, public health care, and health care for persons with disabilities) and include specialized nurses and social workers using a team approach. The final sample consisted of 523 interferential care clients.

### Design and procedure

This study was part of a larger longitudinal project on the effectiveness of interferential care programs in the Netherlands. The data were collected as routine registration within the programs (Routine Outcome Measurement of Monitoring, ROM). Data collection commenced both to aid in the improvement of the program, integrating the new measures into routine registration procedures, and to satisfy research goals, greatly improving comparability to other studies and offering a wider array of outcome measures. Since no special intervention would be implemented merely to aid the needs of the study, and ROM data were used, ethical approval was unnecessary according to the flowchart of the Dutch Medical Ethical Commission (CCMO). However, participants received and read the informed consent form and only those who did not object to the use of their data for research were included in the analyses. Anonymity was assured, and participant data were entered into the system encrypted.

The current study was set up as a cross-sectional study of current SQOL of individuals entering the interferential care program. Therefore, only data collected upon entry into the program were included in our analyses. Participants filled in a self-report SQOL scale in their own living environment. The other observer-rated scales and questions were filled in by the professional caregiver after gathering information from the participant and the referring authority, and sometimes from the social environment of the participant.

### Measurement instruments

*SQOL* was measured using the Dutch version of the Manchester Short Assessment of Quality of Life (Mansa) [19]. This measure was developed as a condensed and modified instrument on the basis of the Lancashire Quality of Life Profile (LQLP) [20]. The properties of the Mansa have been tested in the target population of community care patients. The measure correlates satisfactorily with other SQOL measures, and internal consistency for the satisfaction ratings was 0.74 [20]. The questionnaire contains twelve items asking about satisfaction with life as a whole and satisfaction with several aspects; i.e., work situation, access to resources, quality of friendships, leisure time, living environment, personal safety, the relationship with household members, sexual life, the relationship with other family, physical, and mental health. On the basis of these domain scores, a mean SQOL score can be calculated.

To be able to control for *demographic characteristics*, age, sex, and ethnic background were recorded. For ethnic background, participants stated whether they were native Dutch or of other ethnicity.

*Social environment* was assessed by asking whether the participant has a partner, has any children, or has a good friend. Lastly, two questions were asked on whether the participant had been accused of a felony or whether he or she had been the victim of violence in year prior to the assessment. The latter three items were an integral part of the Mansa.

*Problem severity* of participants was measured using the Health of the Nation Outcome Scales (HoNOS) which is a 12 item widely used routine clinical outcome measure [21]. Training in filling out the HoNOS was provided to the staff member of the ICBC teams at the start of the study, because previous studies had suggested that this greatly improves interrater reliability [21]. Dutch research on psychometric properties of the HoNOS shows that the interrater reliability of the total score was good (0.92) and that the internal consistency varied from 0.78 [21] to 0.64 [22]. The HoNOS distinguishes between hyperactive/aggressive behavior, self-harming behavior, addiction problems, cognitive problems, physical problems, hallucinations/delusions, depressive symptoms, social contact, activities of daily life, living environment, the use of skills, and other clinically relevant behavior. Four subscales can be calculated, being behavior (item 1–3), impairment (item 4–5), symptoms (item 6–8), and social problems (item 9–12).

### Analytical strategy

All analyses were carried out using IBM SPSS Statistics 22©. The basic characteristics of the complete study sample were examined using descriptive statistics. A mean score was calculated for SQOL (Mansa). Separate scores for the HoNOS items were used to calculate total scores for the four subscales and total HoNOS score. To investigate the independent contribution of individual predictors to SQOL, three separate multiple linear regression models were built using a hierarchical procedure.

In the first model, predictors were included and entered in 3 steps. In Step 1, demographic variables were included to be able to control for age, sex, and ethnicity. In Step 2, having a good friend, having a partner, and having children, as well as having been accused of a felony and having been the victim of violence, were included. In Step 3, the total score on the HoNOS, indicating the overall problem severity, was added. The second model was identical to this first model, with the only difference of the inclusion of the domain-based scores on the HoNOS instead of using the total score. The third model was also identical to the first model, with the difference that separates item scores of the HoNOS was included instead of the added total or domain-based subscale scores. The second and third model added to the analyses in that this provided a more detailed picture of the specific problems that were possibly related to (decreased) SQOL.

## Results

### Means and sample characteristics

In the total sample of 523 participants, 345 were male. About a third had children and a minor part had a partner. Most participants had the Dutch nationality. Age ranged from 18 to 86 years, with a mean age of 46. Descriptive statistics of demographics, predictors, and the outcome measure are presented in Table 1.

**Table 1:** Sample characteristics

ICBC clients upon entry into the program (*N* = 523)	
Demographics	
Sex (% male)	66.1 %
Age *M* (SD)	45.7 (15.9)
Ethnicity (% non-native Dutch)	13.6 %
Predictors	
% Having children	37.5 %
% Having a partner	17.7 %
% Having a good friend	71.4 %
% Having met a friend last week	52.6 %
% Having been convicted of a felony in the last year	20.3 %
% Having been a victim of violence in the last year	15.8 %
Problem severity *M* (SD)	14.58 (6.63)
Outcome measure	
Subjective quality of life *M* (SD)	3.85 (.98)

Problem severity was measured by the Health of the Nations Outcome Scale (HoNOS). Score represents the mean of summed HoNOS items. The scale was completed by 518 clients. Quality of life was measured by the Manchester Short Assessment of Quality of Life (Mansa). The scale was completed by 336 clients

The ICBC clients in our study scored significantly higher (*M* = 14.58, SD = 6.63) on problem severity compared to norming scores established in previous research among clients receiving ambulatory mental health care (*M* = 11.2, SD = 7.0, *t* = 11.58, *p* < .000), but scored slightly lower on problem severity compared to a (non-clinical) day treatment setting (*M* = 15.2, SD = 7.3, *t* = −2.14, *p* = .03) and compared to clients in a clinical setting (*M* = 16.1, SD = 7.3, *t* = −5.23., *p* < .000) [21].

### Determinants of quality of life

First, preliminary analyses were conducted to assess analysis assumptions. An analysis of standardized residuals was carried out. The histogram of standardized residuals indicated that the data contained approximately normally distributed errors, as did the normal P–P plot of standardized residuals, which showed points that closely followed the line. The data met the assumption of independent errors (Durbin–Watson model 1 = 1.888, model 2 = 1.862, model 3 = 1.862). The scatterplot of standardized predicted values showed that the data met the assumptions of homogeneity of variance and linearity. Tests to see whether the data met the assumption of collinearity indicated that multicollinearity was not a concern. Tolerances were all over 0.1, and VIF values were all well under 10. The data also met the assumption of nonzero variances.

Secondly, the three hierarchical multiple linear regression models were tested. All hierarchical levels in all three regression models significantly explained variance in ICBC clients’ SQOL (See Table 2). Results are described step-by-step:

**Table 2:** Summary of hierarchical regression analysis for variables predicting SQOL (*N* = 518)

Variable			Model 1				Model 2				Model 3			
			*B*	SE *B*	*β*	*p*	*B*	SE *B*	*β*	*p*	*B*	SE *B*	*β*	*p*
Age			.008	.003	.135*	.015	.005	.003	.083	.142	.003	.004	.052	.365
Sex			−.154	.108	−.075	.154	−.134	.107	−.065	.210	−.189	.107	−.091	.078
Ethnicity			−.280	.142	−.099*	.049	−.272	.139	−.096	.051	−.279	.138	−.099*	.044
Partner			.378	.130	.147**	.004	.340	.127	.132**	.008	.353	.124	.137**	.005
Children			−.253	.109	−.125*	.021	−.245	.107	−.121*	.023	−.164	.106	−.081	.124
Friend			−.580	.108	−.268***	.000	−.549	.106	−.254***	.000	−.534	.105	−.247***	.000
Felony			−.117	.130	−.048	.372	−.123	.132	−.051	.353	−.117	.131	−.048	.372
Victim			−.184	.136	−.068	.179	−.167	.134	−.062	.213	−.201	.131	−.075	.127
HoNOS 1 Aggression	HoNOS Behavioral problems	HoNOS total score	−.050	.007	−.337***	.000	−.051	.022	−.120*	.021	−.063	.042	−.080	.131
HoNOS 2 Self-harm											.048	.082	.029	.553
HoNOS 3 Substance use											−.061	.033	−.095	.063
HoNOS 4 Cognitive problems	HoNOS personal limitations						.004	.025	.008	.888	.037	.038	.048	.331
HoNOS 5 Physical problems											−.006	.039	−.008	.881
HoNOS 6 Psychosis	HoNOS Symptomatology						−.114	.019	−.296***	.000	−.050	.046	−.056	.273
HoNOS 7 Depression											−.281	.042	−.352***	.000
HoNOS 8 Psychiatry/other											−.052	.035	−.078	.144
HoNOS 9 Social contact	HoNOS Social problems						−.037	.013	−.152**	.004	−.024	.041	−.033	.552
HoNOS 10 ADL activities											.002	.039	.002	.996
HoNOS 11 Living conditions											−.092	.030	−.156**	.003
HoNOS 12 Occupation											−.021	.038	−.032	.570
*R* ^2^ change Step 1 Demographics			.033*				.033*				.033*			
*R* ^2^ change Step 2 Social environment			.165***				.165***				.165***			
*R* ^2^ change Step 3 Subjective quality of life			.273***				.313***				.369***			

Having a friend is a negatively formulated item

* *p* < .05, ** *p* < .01; *** *p* < .001

*Step 1 Demographics*: Concerning demographics, none consistently added to the variance explained in all three models. Age was a significant predictor in the first regression model; older individuals indicated a lower SQOL score. Ethnicity was a significant predictor in the first and third model; non-native Dutch clients rated SQOL lower
*Step 2 Social environment*: Of the social variables, having a partner and having a good friend significantly predicted SQOL in all three models; having a good friend and having a partner were positively linked to SQOL. In the first and second model, having children significantly predicted lower SQOL
*Step 3 Problem severity*: When looking at the standardized coefficients of the variables in the three models (including all three steps), problem severity (in model 1, the overall problem severity, in model 2, the HoNOS subscale symptomatology, and in model 3, the separate HoNOS item ‘depressive symptoms’) emerged as the strongest predictor of SQOL, with a decreased problem severity being related to higher SQOL

Total variance explained. Because of the way the HoNOS items were included in the subsequent models, the proportion explained variance can be seen to increase between the first model (total HoNOS score), in which predictors explained 27.3 %, *F*(9, 309) = 12.92, *p* < .001, the second model (HoNOS subscales), in which predictors explained 31.3 %, *F*(12, 306) = 11.60, *p* < .001, and the third model (HoNOS item scores), in which predictors explained 36.9 %, *F*(20, 298) = 8.72, *p* < .001, of the variance in SQOL.

## Discussion

This study explored characteristics of ICBC clients in interferential care programs, as well as the level and determinants of their SQOL based on routinely acquired information upon intake into the program. First and foremost, the results revealed that problem severity (whether total score, subscale score, or separate item score) was a consistent and significant predictor of SQOL. Even after controlling for the demographics, problem severity still explained the largest amount of variance in all three models. When looking at problem severity in the three different models, SQOL is best predicted by the separate items of the HoNOS, indicating that not just overall problem severity, but also the severity of a client’s specific problem types matters for the perceived quality of life. Factors that appear to be highly influential are ‘symptomatology’ (‘depressive symptoms’ to be specific) and ‘social problems’ (‘living conditions’ to be specific). These findings add to previous studies, in which diagnosis and psychopathology were hardly related to SQOL for ICBC clients [11]. In a regular mental healthcare sample, on the contrary, a relation with symptoms was found [14]. This might indicate that ICBC populations are different from regular mental healthcare populations and ICBC populations even differ between regions and countries. Answering this might be key in the question why studies on ICBC show inconsistent results on effectiveness [5]. As Holloway and Carson [23] suggested, ICBC can only be effective if type of ICBC program is matched with the specifics of the target population. Another explanation might be that focusing on the severity (instead of type of psychiatric problems) and inclusion of a wider range of life areas than just psychiatric symptoms give a more precise picture of the relationship between problems and SQOL. Further study to support these assumptions is suggested.

Other influential variables were having a partner and having a good friend. These variables structurally predicted higher SQOL, regardless of which model was used. Although not much is known on the effects of social support on persons with (severe) mental health problems, one study in depressed patients [24] showed that social support can contribute substantially to SQOL. This information could provide tools for healthcare professionals working in ICBC to improve SQOL among their clients. With this finding in mind, it is interesting to see that we find no separate effect of the ‘social contacts’ item in the third model in which separate HoNOS items are included. However, we believe that this could be a result of the HoNOS being rated by the healthcare professionals, whereas the partner and good friend items were self-rated.

An unexpected finding was that ‘having children’ was significantly negatively associated with SQOL (in two of the three models). We believe that a possible explanation for this could be that marginalized people, like ICBC clients, are generally less equipped to care for their children and it might be more difficult for them to maintain good social relationships with them. Therefore, the feeling of being less able to provide care for or maintain social contact with one’s children might explain the negative association between having children and the clients’ SQOL.

Lastly, concerning demographics, non-native Dutch participants rated SQOL lower compared to native Dutch participants as did female compared to male participants.

This study had some strengths and weaknesses. Because data were used that are routinely collected upon intake into the ICBC program, and to make registration feasible, a restricted number of possible determinants could be investigated. This means that there could be other important factors determining quality of life for clients in the ICBC program, which have not been incorporated in this study. Nevertheless, the variables included in this study explained over a third of the variance in SQOL (model 3) and include factors easy to get data on for ICBC team members.

Another small point concerns a possible selection bias, created by the fact that only data from people that voluntarily filled out the SQOL assessment scale (Mansa) were available. Theoretically, these people were possibly either those that were most willing to cooperate with service providers, those that were in the least bad condition, or those that were most satisfied with ICBC in general. This could have affected the results in that the mean measured SQOL might have been higher had a proxy-type measure of SQOL been used. However, this is an unpreventable problem, because quality of life is best assessed by self-report. Furthermore, the response rate was still relatively high for a self-report measure, as in total 64.2 % of the total sample filled out the Mansa. A non-response analysis could have shed light on this issue, but was impossible to conduct due to data restrictions. Future studies could benefit from using both self-report and proxy-type measures for SQOL in the same design to gain more insight into the potential bias that using self-report measures regarding this topic might induce.

Lastly, although our study adds interesting and important new knowledge, our study is still only an indication of SQOL and its determinants for clients receiving ICBC. Unfortunately, the methodology used did not allow going beyond an exploration of the determinants. We argue that in the future, qualitative research would be of added value in order to complement the results of the present study.

The present study has a number of strengths. A first strength of this study includes the large total number of included participants, enlarging the reliability and generalizability of the results. A second strength is that the study takes into account problem severity in several specific life areas that could be related to SQOL, instead of focusing on psychopathology only. Moreover, effects of the social environment were included in the models as well. Lastly, the broad scope of this study has proven to be one of its strengths. It has provided us with very important new knowledge; having social contacts (a partner and/or a good friend), next to depressive symptoms and living conditions, appears to be the strongest predictors of SQOL.

The findings of this study provide new insights into ICBC clients’ self-perceived quality of life. SQOL in the ICBC target population does seem to be related to psychopathology, in contrast to previous knowledge. However, it also appears to be dependent upon specificity of symptoms, living conditions, and social circumstances and therefore presumably on ICBC program characteristics.

The current study reveals that problem severity is an important predictor of the perceived quality of life of ICBC clients receiving ICBC care from an interferential care team. This knowledge adds to our understanding of what well-being consists of for these people and is thereby relevant to healthcare professionals working with the target group in practice.

Several recommendations to healthcare professionals and program developers are justified. First of all, this study suggests that the greatest amount of progress within the program might be established by working toward an improvement in depressive symptoms, as well as living conditions of the person. These problem areas therefore deserve the utmost attention from professional caregivers working with ICBC clients. Additionally, the established relationship of SQOL with social environment also asks for special attention of ICBC caregivers, as not having either a partner or a good friend appears to be risk factors for a lower SQOL. Assistance in creating a good social support system could be a task for healthcare providers that could greatly improve outcomes for ICBC clients. In conclusion, the findings of this study could be relevant in answering the question of what type of ICBC program is required to best suit each individual ICBC client’s needs.
